# Targeted massively parallel sequencing panel to diagnose genetic endocrine disorders in a tertiary hospital

**DOI:** 10.1016/j.clinsp.2022.100132

**Published:** 2022-10-23

**Authors:** Amanda M. Narcizo, Lais C. Cardoso, Anna F.F. Benedetti, Alexander A.L. Jorge, Mariana F.A. Funari, Barbara L. Braga, Monica M. Franca, Luciana R. Montenegro, Antonio M. Lerario, Mirian Y. Nishi, Berenice B. Mendonca

**Affiliations:** a Laboratório de Sequenciamento em Larga Escala (SELA), Faculdade de Medicina da Universidade de São Paulo, São Paulo, Brazil; b Laboratório de Hormonios e Genetica Molecular/LIM42, Hospital das Clínicas, Faculdade de Medicina da Universidade de São Paulo, São Paulo, Brazil; c Unidade de Endocrinologia Genetica/LIM25, Faculdade de Medicina da Universidade de São Paulo, São Paulo, Brazil; d Department of Internal Medicine, Division of Endocrinology, Metabolism and Diabetes, University of Michigan, Ann Arbor, MI, USA

**Keywords:** Multigenic custom panel, Endocrine disorders diagnostic

## Abstract

**Objectives:**

To analyze the efficiency of a multigenic targeted massively parallel sequencing panel related to endocrine disorders for molecular diagnosis of patients assisted in a tertiary hospital involved in the training of medical faculty.

**Material and methods:**

Retrospective analysis of the clinical diagnosis and genotype obtained from 272 patients in the Endocrine unit of a tertiary hospital was performed using a custom panel designed with 653 genes, most of them already associated with the phenotype (OMIM) and some candidate genes that englobes developmental, metabolic and adrenal diseases. The enriched DNA libraries were sequenced in NextSeq 500. Variants found were then classified according to ACMG/AMP criteria, with Varsome and InterVar.

**Results:**

Three runs were performed; the mean coverage depth of the targeted regions in panel sequencing data was 249×, with at least 96.3% of the sequenced bases being covered more than 20-fold. The authors identified 66 LP/P variants (24%) and 27 VUS (10%). Considering the solved cases, 49 have developmental diseases, 12 have metabolic and 5 have adrenal diseases.

**Conclusion:**

The application of a multigenic panel aids the training of medical faculty in an academic hospital by showing the picture of the molecular pathways behind each disorder. This may be particularly helpful in developmental disease cases. A precise genetic etiology provides an improvement in understanding the disease, guides decisions about prevention or treatment, and allows genetic counseling.

## Highlights

• The multigenic panel effectively diagnoses patients with endocrine diseases.
• It is the first panel to diagnose a broad spectrum of endocrine diseases using TMPS.
• This panel helps the visualization of molecular pathways behind endocrine conditions.

## Introduction

The diagnostic approach to endocrine diseases has traditionally been based on a set of physical findings, biochemical testing, and imaging studies.^1^ With advances in new sequencing technologies, the role of genetic diagnosis in endocrinology has been transforming its methodology, allowing genetic testing to assume an exploratory role rather than solely a confirmatory one. In this sense, the availability of Next-Generation Sequencing (NGS) allowed the identification of novel candidate genes additionally to an in-depth modification of the understanding of the architecture of several diseases.^2^

In routine diagnostics, detecting pathogenic variants via conventional Sanger sequencing is still the standard, despite the practical difficulties of keeping up with the ever-increasing numbers of test requests and disease-associated genes.^3^ Sanger only allows the analysis of one DNA segment at a time and is laborious, expensive, and time-consuming.^4^ However, the transition from Sanger sequencing to high-throughput massively parallel sequencing technologies enabled the analysis of multiple regions of a genome in a single reaction, decreasing the costs and time involved in obtaining high-quality DNA sequence data to investigate patients with endocrine diseases.^1^

In endocrine disorders, the complex and intricate genotype-phenotype relations and occurrence of diverse comorbidities led to a challenging investigation. A way to further increase efficiency and sensitivity for pathogenic variants detection is the use of a sequencing custom panel selecting specific genes for screening.^5^

The present study's aim is to analyze the efficiency of a multigenic panel related to endocrine disorders for molecular diagnosis of undiagnosed patients assisted in a tertiary hospital with suspected endocrine diseases that may carry a genetic variant capable of justifying their phenotype.

## Patients and methods

272 patients affected with different endocrine disorders treated at Hospital das Clínicas da Faculdade de Medicina da Universidade de São Paulo (HCFMUSP), São Paulo, Brazil were selected. The patients were divided depending on their main clinical presentations, falling into 1 of 5 categories: developmental, metabolic, adrenal, thyroid or neuroendocrine diseases. The Sanger method was used to screen candidate genes in half of the patients before doing the next-generation sequencing.

This study was conducted in accordance with the Declaration of Helsinki and it was approved by the Research Ethics Committee of the FMUSP under protocol number 4.609.656.

### Targeted massively parallel sequencing (TMPS) and analysis

To start the TMPS process the genomic DNA was extracted in 272 patients from peripheral blood leukocyte samples using the salt precipitation method.^6^ The patients were divided depending on their main clinical presentations, falling into 1 of 5 categories: developmental, metabolic, adrenal, thyroid, or neuroendocrine diseases.

A custom panel for diagnostic endocrine diseases was designed containing 653 genes using the SureDesign tool (Agilent Technologies, Santa Clara, CA, USA). Out of these 653 genes, 640 were found in OMIM (Online Mendelian Inheritance in Man) database. Also, 24 of the 653 genes are reported by ACMG as obligatory reports in case of secondary findings.^7^ A list of all the genes analyzed can be found in the Supplementary files.

The DNA was sheared into short fragments using the Covaris E220 adaptive focused acoustics instrument (COVARIS, INC., Woburn, MA, USA). PCR enrichment and hybridization of the fragments were prepared with SureSelectXT Target Enrichment kit (Agilent Technologies, Santa Clara, CA, USA) according to the protocol in Bravo Automated Liquid Handling Platform (Agilent Technologies, Santa Clara, CA, USA). The enriched DNA libraries were sequenced in NextSeq 500 platform (Illumina, Inc., San Diego, CA, USA) with High Output V2 kit and paired-end (2×150 bp) sequencing reads. This sequencer is part of the PREMiUM Multi-User Equipment Network (Large Scale Sequencing Laboratory) of the School of Medicine of Universidade de São Paulo.

To guarantee sufficient data quality, the number of samples per sequencing was calculated to obtain a minimum of 20× depth of coverage. The raw reads were preprocessed and aligned to reference genome GRCh 37/hg 19 version of the human genome through BWA-MEM tool,^8^ variant calling was performed using FreeBayes.^9^ and functional genetic variants were annotated with ANNOVAR software.^10^

**Fig. 1:**
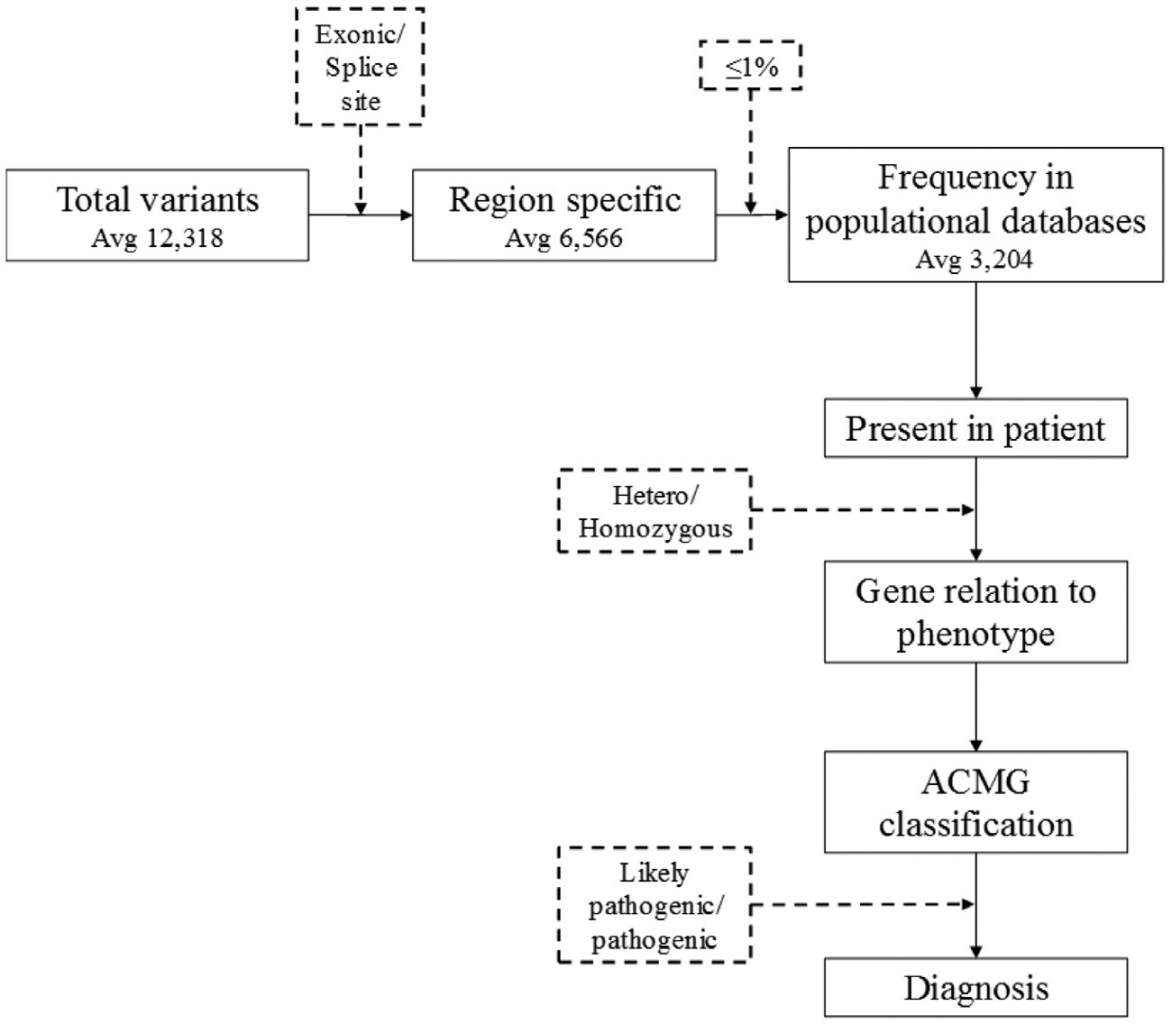
Filtering of the gene panel for each patient in order to reach diagnosis.

Filtering took into consideration those located in exonic or splice site regions and the rarity (≤1%) of variants in international population databases (gnomAD and 1000 genomes), as well as an internal national database (SELAdb)^11^ (Fig. 1). Variants that were found were then classified according to ACMG/AMP criteria,^7^ with the aid of Varsome^12^ and InterVar^13^ tools. Thus, the authors reached a list of variants that are classified as 1) Pathogenic (P): variants that alter the protein functions and may have been previously reported in other affected individuals, 2) Likely Pathogenetic (LP): contribute to the development of disease, however, the scientific data are insufficient to confirm the pathogenicity, and 3) Uncertain Significance (VUS): are variants that could possibly affect protein function based on results from *in silico* software prediction tools.^5^ Only variants classified as pathogenic or likely pathogenic are considered a “genetic diagnosis” in accordance with guidelines^7^ while Variants of Uncertain Significance (VUS) were only reported.

## Results

The 272 patients recruited for the study were sequenced in three independent runs. The average of the run metrics of the three runs was 76.6 Gb (SD ± 6.5) of data, cluster density of 159 Kmm^−2^ (SD ± 19.9) and 76.5% (SD ± 63.4) of bases had a quality score (Q) of 30.

The three runs produced mean total reads of 6,250,746.248 and 6,218,365.715 mapped reads, which met the Q30 quality criteria meaning that only reads were included in which the error probability for each base has a likelihood of 1/1,000. Quality metrics were assessed to validate the performance of multigenic panels. In this analysis, an average sequencing depth of 249× on targeted regions and at least 96.9% of the sequenced bases were covered by twenty or more reads. The overview of the sequence performance of three runs is displayed in Table 1.

**Table 1:** Average of each run and of all three runs for each quality assessment.

Quality Assessment	Mean of runs
Q30 (%)	76.5
Yield (Gb)	76.6
Cluster density (Kmm^−2^)	159
Total reads	6,250,746.2
Mapped reads	6,218,365.7
Mean coverage of target	249
% of target with >20×	96.9

Q, Quality score; Gb, Gigabases; Kmm^−2^, 1000 (K) clusters per square millimeter (mm^2^).

**Fig. 2:**
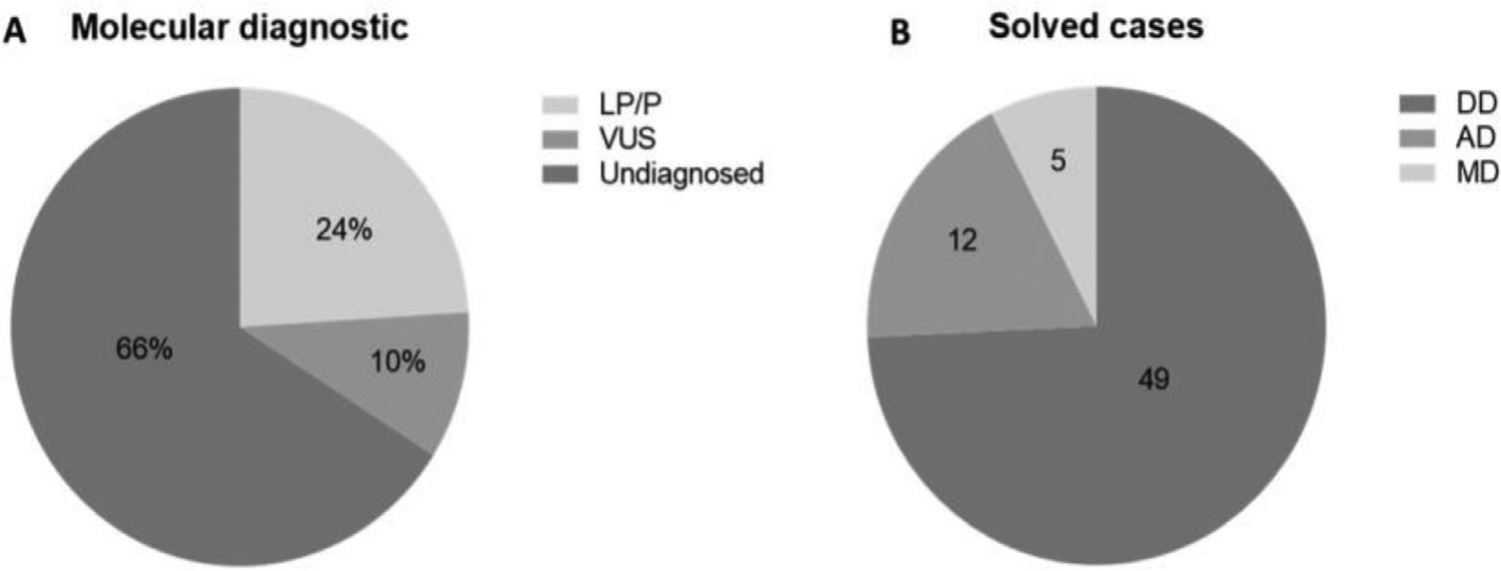
(A) Molecular diagnosis in 272 patients, 24% (66) was diagnostic as LP/P, 10% (27) were VUS and 66% (179) were undiagnosed. (B) Solved cases. Of the 66 cases solved, 49 (74.2%) have Developmental Diseases (DD), 12 (18.2%) have Metabolic (MD) and 5 (7.6%) have Adrenal Diseases (AD).

Out of the 272 patients who took part in the study, a molecular diagnosis was identified in 93 patients (34%). Only Pathogenic (P) or Likely Pathogenetic (LP) variants, identified in 66 patients (24%) were considered for molecular diagnosis. Variants of Uncertain Significance (VUS) were found in 27 (10%) patients (Fig. 2A).

## Discussion

The Targeted Massively Parallel Sequencing (TMPS) multigenic panel proved to be efficient in the diagnosis of patients with endocrine diseases undergoing investigation by TMPS. The total number of diagnosed cases was 24%, of which 74.2% are developmental disease cases, 18.2% metabolic, and 7.6% adrenal. The authors’ findings are similar to other published data. A study that used a target panel to sequence 80 genes, including known genes associated with human Disorders of Sex Development (DSD) and some recently identified genes that influence the pathways of sex development obtained a diagnostic rate of 28%^14^; this result was similar two other DSD studies (29% and 22.5%).^15^^,^^16^ Also, the authors identified 10%  of Variants of Uncertain Significance (VUS) that warrant further studies to determine their relation to the patient's clinical findings. Lastly, 66% of patients only presented benign variants.

The multigenic panel was the first one described in the literature for the diagnosis of a large spectrum of endocrine diseases by targeted massively parallel sequencing. In this study half of the patients had already been previously screened to diagnostic methodologies for the main candidate genes for their phenotypes. Besides that, the analysis of the multigenic panel allowed the development of research in the different fields of endocrinology in the present study's hospital. Molecular diagnosis was possible in 33% of the patients with 46, XY gonadal dysgenesis, and NR5A1 variants were the most prevalent molecular defects.^17^ Through these studies, it was possible to identify a new mutation and a deletion of the entire coding sequence in genes related to Congenital Hypogonadotropic Hypogonadism (CHH).^18^ In short Stature Homeobox (SHOX) haploinsufficiency studies, the target panel confirmed changes in known genes and identified two new deletions that were not previously detected through the classical diagnosis, the MPLA followed by Sanger.^19^ In patients with congenital GH deficiency, the target panel was an efficient method to simultaneously screen variants of biological and clinical relevance. A genetic diagnosis was possible in 5 out of 117 (4%) patients of the present study's cohort. The authors identified three novel pathogenic variants in GHRHR, OTX2, and GLI2 expanding the spectrum of variants associated with congenital hypopituitarism.^20^ In addition, the target panel can be used for the molecular investigation of patients with Idiopathic Short Stature (ISS).^21^ Another study carried out a genetic analysis using a personalized DNA target enrichment panel that made it possible to identify several rare dominant variants in genes implicated in the migration and metabolism of GnRH in Constitutional Growth and Delayed Puberty (CDGP).^22^

In addition, this process was crucial for the present study's medical and biochemistry students and fellows to familiarize themselves with this new diagnostic methodology and expand their knowledge of molecular biology as a whole.

Analysis of the data enabled an expansion of the understanding of gene function and allowed us to identify genes with known roles in pathology and to associate them with other clinical conditions. Recent advances in DNA sequencing technologies have resulted in an intense period of disease‐gene discovery, and greater insight into the molecular‐genetic basis of many endocrine disorders.^23^^,^^24^

The targeted massively parallel sequencing application is widely used to identify gene alterations that are targetable by molecular‐targeted drugs,^25^ identify novel pathogenic mechanisms,^2^ and provide early diagnosis of individuals of the same family.^26^ It's a cost-effective solution adopted in a great number of laboratories allowing the optimization of different features of molecular diagnostics workflow (reduced costs for library preparation steps and sequencing run, adaptability to different kinds of samples, increased number of samples processed in each run and reduction of time needed for clinical counseling).^27^ Another advantage of custom target sequencing is the possibility to personalize the panel (i.e., the inclusion of certain genes and the possibility to sequence exons, specific intronic regions, promoter regions, or the 3′ untranslated regions).^5^ Furthermore, targeted massively parallel sequencing often has demonstrably better sensitivity for mutations than whole exome protocols.^28^ Besides that, the application of a multigenic panel aids the training of medical faculty in an academic hospital by showing the big picture of the molecular pathways behind each disorder. This may be particularly helpful considering the higher diagnosis of developmental disease cases. A precise genetic etiology provides an improvement in understanding the disease, guides decisions about prevention or treatment, allows genetic counseling, and provides a clear explanation of the cause for the patient and family.

## Authors' contributions

Narcizo AM and Cardoso LC were responsible for writing and data generation. Benedetti AFF was responsible for the data analysis. Funari MFA, Braga BL, Franca MM, Montenegro LR, Nishi MY were responsible for the data generation. Lerario AM was responsible for data analysis and supervision. Jorge AAL and Mendonça BB were responsible for the project coordination, supervision, and manuscript editing.

## Declaration of Competing Interest

The authors declare no conflicts of interest.

## Appendix Supplementary materials

Image, application 1
