# Supplementary material for: Targeted massively parallel sequencing panel to diagnose genetic endocrine disorders in a tertiary hospital
Source: Clinics (Sao Paulo). 2022 Oct 23;77:100132. doi: 10.1016/j.clinsp.2022.100132 (PMC9593712; doi:10.1016/j.clinsp.2022.100132)
Supplement: Supplementary file 1 [file mmc1.docx]

**CLINICS-D-22-00486 – Supplementary Material**

**Supplementary files**

List of all the genes analyzed

| A2ML1 | APPL1 | BSCL2 | CKS2 | DMC1 | FBN2 | GHR |
| --- | --- | --- | --- | --- | --- | --- |
| AAAS | AQP2 | BTK | CLCN7 | DMP1 | FBP1 | GHRH |
| ABCA1 | AR | C2ORF80 | COL1A1 | DMRT1 | FEZF1 | GHRHR |
| ABCC8 | ARL6 | CACNA1D | COL1A2 | DMRT2 | FGD1 | GHRL |
| ABCD1 | ARMC5 | CACNA1H | COL2A1 | DMXL2 | FGF17 | GHSR |
| ABCG1 | ARNT2 | CADM1 | COL4A6 | DNA2 | FGF23 | GJA1 |
| ACADVL | ARX | CAMK1D | COL9A2 | DNAJC15 | FGF8 | GJA4 |
| ACAN | ATG7 | CARTPT | COL9A3 | DND1 | FGF9 | GLI2 |
| ACBD7 | ATM | CASP8 | COMP | DNMT3A | FGFR1 | GLI3 |
| ACP5 | ATP1A1 | CASR | CP | DUOX2 | FGFR2 | GLIS3 |
| ACVR1B | ATP2B3 | CAV1 | CPEB1 | DUOXA2 | FGFR3 | GLUD1 |
| ACVRL1 | ATR | CBL | CREBBP | DUSP6 | FH | GNA11 |
| ADAMTS10 | ATRIP | CBLN1 | CRIPT | DYRK1B | FIGLA | GNAS |
| ADAMTS16 | ATRX | CBS | CRTAP | EBF2 | FKBP10 | GNRH1 |
| ADAMTS17 | AVP | CBX2 | CTNNB1 | EGLN1 | FKBP4 | GPBAR1 |
| ADAMTS19 | AVPR2 | CCDC141 | CTSK | EIF2AK3 | FLNB | GPC3 |
| AGL | AXL | CCDC8 | CUL3 | EIF2B2 | FLRT3 | GPIHBP1 |
| AGPAT2 | BAP1 | CDC25B | CUL7 | EIF2B5 | FMN2 | GPR101 |
| AGRP | BBS1 | CDC6 | CYP11A1 | EIF4ENIF1 | FMR1 | GPR161 |
| AIP | BBS10 | CDC73 | CYP11B1 | EIF4G2 | FOXL2 | GPR3 |
| AIRE | BBS12 | CDK2 | CYP11B2 | EMX2 | FOXO1 | GPR83 |
| AKR1C2 | BBS2 | CDKN1A | CYP17A1 | ENPP1 | FOXO3 | GSK3B |
| AKR1C3 | BBS4 | CDKN1B | CYP19A1 | EP300 | FOXO4 | GTF2F1 |
| AKR1C4 | BBS5 | CDKN1C | CYP1B1 | EPAS1 | FOXP3 | GYS2 |
| AKT1 | BBS7 | CDKN2B | CYP21A2 | ERCC1 | FRAT1 | H6PD |
| AKT2 | BBS9 | CDKN2C | CYP24A1 | ERCC2 | FSHR | HABP2 |
| ALMS1 | BCL2 | CDON | CYP26B1 | ERCC6-PGBD3 | FST | HADH |
| ALPL | BCL2L2 | CDT1 | CYP27B1 | ESR1 | G6PC | HARS2 |
| AMH | BLK | CEL | CYP2R1 | EVC | GADD45G | HDAC8 |
| AMHR2 | BLM | CENPJ | DACH2 | EVC2 | GALNT3 | HELQ |
| ANGPTL4 | BMP1 | CEP152 | DAZL | EZH2 | GATA3 | HESX1 |
| ANKRD11 | BMP15 | CEP19 | DBH | FAM111A | GATA4 | HFM1 |
| AP2S1 | BMP2 | CEP63 | DGCR8 | FAM189A2 | GATA6 | HHAT |
| APC | BMP4 | CETP | DHCR7 | FAM20C | GCG | HHIP |
| APOA1 | BMP8B | CHD7 | DHH | FANCA | GCK | HK3 |
| APOA5 | BMPR1B | CHM | DHX37 | FANCC | GCM2 | HMGA1 |
| APOB | BMPR2 | CIDEC | DIAPH2 | FANCG | GDF5 | HMGA2 |
| APOC2 | BRAF | CISD2 | DICER1 | FANCL | GDF9 | HNF1A |
| APOC3 | BRSK1 | CITED2 | DKK1 | FBLN2 | GDNF | HNF1B |
| APOE | BRWD1 | CITED4 | DLK1 | FBN1 | GH1 | HNF4A |

| HPGDS | KISS1 | MATN3 | NOBOX | PDGFB | PTGDS | SERPINH1 |
| --- | --- | --- | --- | --- | --- | --- |
| HRAS | KISS1R | MAX | NOS3 | PDGFRA | PTH | SGOL2 |
| HS6ST1 | KIT | MC2R | NOTCH2 | PDGFRB | PTH1R | SHANK3 |
| HSD11B1 | KITLG | MC4R | NPPC | PDX1 | PTHLH | SHH |
| HSD11B2 | KL | MCM4 | NPR2 | PEX1 | PTPN11 | SHOC2 |
| HSD17B3 | KLF11 | MCM8 | NPR3 | PEX7 | PTRF | SHOX |
| HSD17B4 | KLHL3 | MCM9 | NR0B1 | PGR | PUM2 | SIM1 |
| HSD3B2 | KLLN | MECP2 | NR0B2 | PGRMC1 | RAC1 | SIRT1 |
| HSF2 | KMT2D | MED12 | NR2F2 | PHEX | RAD21 | SIX1 |
| HYMAI | KRAS | MEI1 | NR3C1 | PIK3CA | RAD51B | SIX4 |
| IER3IP1 | LARS2 | MEN1 | NR5A1 | PIK3R1 | RAF1 | SLC16A1 |
| IFITM5 | LCAT | MET | NRAS | PITX2 | RASA2 | SLC16A2 |
| IGF1 | LDLR | MKKS | NRIP1 | PLAG1 | RBBP8 | SLC19A2 |
| IGF1R | LDLRAP1 | MKRN3 | NSD1 | PLAGL1 | REC8 | SLC26A2 |
| IGF2 | LEF1 | MLH1 | NSMF | PLIN1 | RET | SLC26A4 |
| IGFALS | LEP | MLH3 | NUP107 | PLK4 | RFX6 | SLC2A2 |
| IGSF1 | LEPR | MNX1 | NXF5 | PLS3 | RIT1 | SLC34A1 |
| IGSF10 | LEPRE1 | MOS | OBSL1 | PMS2 | RNF216 | SLC34A3 |
| IHH | LGR5 | MRAP | OLAH | PNPLA6 | RNPC3 | SLC37A4 |
| IKBKB | LHCGR | MSH4 | ORC1 | POF1B | RNU4ATAC | SLC5A5 |
| IL17RD | LHX1 | MSH5 | ORC4 | POLD1 | ROR2 | SLC9A3R1 |
| INHA | LHX3 | MSX1 | ORC6 | POLR3A | RRAS | SMAD1 |
| INHBA | LHX4 | MTHFR | OSTM1 | POLR3B | RSPO1 | SMAD4 |
| INHBB | LHX8 | NANOS1 | OTUD4 | POLR3H | RSPO2 | SMAD5 |
| INS | LHX9 | NANOS2 | OTX2 | POMC | SAMD9 | SMARCE1 |
| INSL3 | LIPC | NANOS3 | P4HB | POR | SCARB1 | SMC1A |
| INSR | LIPE | NBN | PAPPA | POU1F1 | SCNN1A | SMC1B |
| IRS1 | LMNA | NCOA1 | PAPPA2 | POU5F1 | SCNN1B | SMC3 |
| IRS2 | LMNB2 | NCOA2 | PAPSS2 | PPARG | SCNN1G | SNURF |
| IRX3 | LPL | NCOA3 | PAX4 | PPIB | SDHA | SOCS2 |
| IYD | LRP5 | NCOA4 | PAX6 | PRDM1 | SDHAF2 | SOHLH1 |
| KAL1 | LTBP2 | NDUFB3 | PAX8 | PRKACG | SDHB | SOHLH2 |
| KATNAL1 | LTBP3 | NEUROD1 | PC | PRKAR1A | SDHC | SOS1 |
| KATNAL2 | LZTR1 | NEUROG3 | PCK1 | PRLR | SDHD | SOS2 |
| KATNBL1 | MAMLD1 | NF1 | PCNT | PROK2 | SEC24D | SOST |
| KCNJ11 | MAP2K1 | NFIX | PCSK1 | PROKR2 | SECISBP2 | SOX10 |
| KCNJ5 | MAP2K2 | NIN | PCSK9 | PROP1 | SEMA3A | SOX13 |
| KCNQ1OT1 | MAP3K1 | NIPBL | PCYT1A | PSMC3IP | SEMA3E | SOX2 |
| KDM6A | MAP3K4 | NKX2-2 | PDE11A | PTEN | SEMA7A | SOX3 |
| KIF1B | MAP4 | NNT | PDGFA | PTF1A | SERPINF1 | SOX7 |

| SOX8 | TGFBR3 | WT1 |
| --- | --- | --- |
| SOX9 | THRA | WWOX |
| SP7 | THRB | XPNPEP2 |
| SPARC | TIAL1 | XRCC4 |
| SPINK5 | TKT | ZBTB20 |
| SPO11 | TMEM127 | ZFP57 |
| SPR | TMEM38B | ZFPM2 |
| SPRY4 | TNFRSF11A | ZFX |
| SQSTM1 | TNFRSF11B | ZGLP1 |
| SRA1 | TOP3B | ZMPSTE24 |
| SRCAP | TP53 |  |
| SRD5A1 | TPO |  |
| SRD5A2 | TRIM32 |  |
| SRY | TRIM37 |  |
| STAG3 | TRIP13 |  |
| STAR | TRPS1 |  |
| STAT3 | TSHB |  |
| STAT5B | TSHR |  |
| STIM1 | TTC8 |  |
| STK11 | UBB |  |
| STPG1 | UBE3A |  |
| STRA8 | UBR2 |  |
| STX16 | UCP1 |  |
| SULT2A1 | UCP2 |  |
| SUPT3H | UCP3 |  |
| SYCE1 | USP34 |  |
| SYCP1 | USP9X |  |
| SYCP2 | VDR |  |
| SYCP3 | VHL |  |
| TAC3 | VPS13B |  |
| TACR3 | WDR11 |  |
| TAF4B | WFS1 |  |
| TBC1D4 | WNK1 |  |
| TBCE | WNK4 |  |
| TBX1 | WNT1 |  |
| TCF21 | WNT3A |  |
| TCIRG1 | WNT4 |  |
| TDRD1 | WNT5A |  |
| TG | WNT9B |  |
| TGFB3 | WRN |  |
